# Association study of promoter polymorphisms at the dopamine transporter gene in Attention Deficit Hyperactivity Disorder

**DOI:** 10.1186/1471-244X-9-3

**Published:** 2009-02-05

**Authors:** Xiaohui Xu, Jonathan Mill, Bo Sun, Chih-Ken Chen, Yu-Shu Huang, Yu-Yu Wu, Philip Asherson

**Affiliations:** 1 MRC Social, Genetic and Developmental Psychiatry Centre, Institute of Psychiatry, King's College London, UK; 2 School of Medicine, King's College London, UK; 3 Department of Psychiatry, Chang Gung Memorial Hospital, Taiwan; 4 Chang Gung University School of Medicine, Taiwan; 5 Department of Child Psychiatry, Chang Gung Children's Hospital, Taiwan

## Abstract

**Background:**

Attention deficit hyperactivity disorder (ADHD) is a complex neurobehavioral disorder. The dopamine transporter gene (DAT1/*SLC6A3*) has been considered a good candidate for ADHD. Most association studies with ADHD have investigated the 40-base-pair variable number of tandem repeat (VNTR) polymorphism in the 3'-untranslated region of DAT1. Only few studies have reported association between promoter polymorphisms of the gene and ADHD.

**Methods:**

To investigate the association between the polymorphisms -67A/T (rs2975226) and -839C/T (rs2652511) in promoter region of DAT1 in ADHD, two samples of ADHD patients from the UK (n = 197) and Taiwan (n = 212) were genotyped, and analysed using within-family transmission disequilibrium test (TDT).

**Results:**

A significant association was found between the T allele of promoter polymorphism -67A/T and ADHD in the Taiwanese population (*P *= 0.001). There was also evidence of preferential transmission of the T allele of -67A/T polymorphism in combined samples from the UK and Taiwan (*P *= 0.003). No association was detected between the -839C/T polymorphism and ADHD in either of the two populations.

**Conclusion:**

The finding suggests that genetic variation in the promoter region of DAT1 may be a risk factor in the development of ADHD.

## Background

Attention deficit hyperactivity disorder (ADHD) is a common and highly heritable neurodevelopmental disorder that affects 3–10% of children and 2–4% of adults [1,2]. ADHD is characterized by inattention, hyperactivity and impulsivity. Data from twin, adoption and family studies show that ADHD is highly heritable, and that genetic variation is likely to play a substantial role in the etiology of the disorder [3]. Molecular genetic and pharmacological studies suggest the involvement of the dopaminergic, serotonergic and noradrenergic neurotransmitter systems in the pathogenesis of ADHD. Polymorphic variants in several genes involved in regulation of the dopamine, and related neurotransmitter pathways have been reported to be associated with ADHD [3-6].

The dopamine transporter (DAT) plays a key role in the regulation of dopaminergic neurotransmission by mediating the active reuptake of synaptic dopamine. Several lines of evidence implicate altered function of DAT in the aetiology of ADHD. In particular, the DAT is a major target for various pharmacologically active stimulants, including methylphenidate, which is one of the most commonly prescribed treatments for ADHD.

Functional variants in the DAT1 gene have been widely studied in aetiological studies of ADHD. Most association studies between DAT1 and ADHD have concentrated on a variable number of tandem repeat (VNTR) polymorphism in the 3'-untranslated region of the gene. Cook et al. [7] first reported an association between the 10-reapeat allele of this polymorphism and ADHD, and several groups subsequently replicated this finding [8-13], although such replications are not ubiquitous and several studies have failed to detect any association [14-21]. More recent studies have examined additional markers across the DAT1 gene, finding stronger evidence for an association with multi-marker haplotypes containing the 10-repeat VNTR allele [18,22]. The 10-repeat allele has been associated with quantitative ADHD-trait scores in general population samples [23,24], suggesting it may act as a quantitative trait locus (QTL) influencing hyperactivity in the normal range above and beyond its role as a risk for clinical ADHD. The mechanism behind the reported associations is not yet understood, although several lines of evidence implicate variation in gene expression [25,26].

Linkage disequilibrium does not extend across the entire *DAT1 *gene, and recent evidence suggests the presence of independent risk variants at the 5' end of the gene. Rubie et al. [27] found four polymorphisms (-67A/T, -839C/T, -1169C/G, -1476T/G), all in tight LD in the DAT1 promoter region that are related to potential transcriptional recognition sites. It is plausible that these polymorphisms may be functional and thus potential candidates for a role in ADHD. Recently, three studies have investigated several of these promoter polymorphisms in clinical ADHD samples [20,28,29]. In a large study of 51 candidate genes, Brookes et al [28] found nominal significance with one or more SNPs in 18 genes, including -839C/T polymorphism of DAT1, with the C allele being significantly over-transmitted to affected ADHD probands (χ^2 ^= 5.58, *P *= 0.018). Genro et al. [20] also investigated this SNP in a sample of 243 Brazilian ADHD children and adolescents, and also found a significant association for biased transmission of the C allele to ADHD children (*P *= 0.03). Another study examined the association between the DAT1 promoters -67A/T polymorphism and ADHD in a case/control study, finding a significant association with the T allele [29].

In this study we aimed to provide further clarification of reported associations by genotyping two polymorphisms in the DAT1 promoter region for association with ADHD in two independent family-based clinical samples from the UK and from Taiwan.

## Methods

### Sample characteristics

For the UK sample DNA was collected using buccal swabs from 197 ADHD combined subtype probands, from both parents in 133 families, and from only the mother in 64 families. One hundred and seventeen of the ADHD probands had at least one sibling who was also genotyped. Cases were referred for assessment if they were thought by experienced clinicians to have a diagnosis of the combined subtype of ADHD under DSM-IV criteria, with no significant Axis I co-morbidity apart from oppositional defiant disorder (ODD) and conduct disorder (CD) and IQ greater than 70. The age range was 5–15 years at the time of assessment (mean 10.41, SD 2.34). Parents were interviewed with a modified version of the Child and Adolescent Psychiatric Assessment (CAPA) [30]. Information on ADHD symptoms at school was obtained using the long form of the Conner's questionnaire [31]. The subjects gave their written informed consent, and this study was approved by the Ethical Committee of King's College London (Reference number: G9814668).

The Taiwanese sample consisted of 212 children with ADHD diagnosed between the ages of 5–15 years (mean 8.96, SD 2.60). Both parents were available for 114 families, only the mother for 59 families and only the father for 39 families. ADHD cases were ascertained from the Child Psychiatric Clinics in the Chang Gung Memorial Hospital in Taipei area, Taiwan. A diagnosis of ADHD was made according to DSM-IV criteria following completion of a standard maternal interview [32], and completion of parent and teacher Conner's revised rating scales [31]. Autism cases were excluded from the study. No other neurological or behavioural disorders were identified. Subjects gave their written informed consent, and were approved by the Institutional Review Board, Chang Gung Memorial Hospital, Taiwan (Reference number: 94-471 and 96-0058B).

### Molecular genetics

The -67A/T (rs2975226) and -839C/T (rs2652511) polymorphisms were genotyped using PCR and enzyme digestion. For the -67A/T SNP the genomic DNA was amplified using forward primers 5'CCAGCCATCTGCGTCC-3' (spanning nucleotide position -151 to -135) and reverse primers 5'-GATGCCGAGAGCGACG-3' (spanning nucleotide position +98 to +113). For the -839C/T SNP the PCR was performed using forward primers 5'GCTCACGGGAGCATCGAG-3' (spanning nucleotide position -973 to -956) and reverse primers 5'-GCACTCGCCTAAGAAAACCA-3' (spanning nucleotide position -689 to -708). After amplification of genomic DNA the PCR product were digested with restriction enzyme Tth111I (for -67A/T SNP) and MspI (for -839C/T SNP) (New England Biolabs). The detail protocols were described in previous study [27].

### Statistical analysis

Family genotype data were analysed using the transmission disequilibrium test (TDT) implemented in UNPHASED program (TDTPHASE) [33], . Linkage disequilibrium (LD) was estimated by calculating D' and r^2 ^statistics. The Bonferroni correction was applied for multiple comparisons and *P *< 0.0125 was considered to show a statistically significant difference.

## Results

Population frequencies for the two markers were estimated from parental genotypes. For the UK and the Taiwanese sample the A-allele frequency of the -67A/T polymorphism is 61% and 68%, respectively, and the C-allele frequency of the -839C/T polymorphism is 52% and 53%, respectively. Allele frequencies of both markers did not show any significant deviation from those expected according to Hardy -Weinberg equilibrium in either population. Significant LD was observed between the two markers (D' = 1, r^2 ^= 0.91 in UK samples; D' = 0.70, r^2 ^= 0.59 in Taiwanese samples).

TDT analysis (Table 1) showed that the T allele of the -67A/T polymorphism was significantly over-transmitted to affected probands in the Taiwanese population (χ^2 ^= 10.43, *P *= 0.001, odds ratio = 1.8). Even after correcting *P*- values using the Bonferroni method for multiple comparisons a significant association was still found between the -67T allele and ADHD cases in Taiwanese population. No significant association was seen in the UK sample for this marker using either standard TDT analysis, or the extended TDT analysis including siblings, although the T-allele was over-transmitted (OR = 1.16). A combined analysis including both population samples found significant association of the T-allele (χ^2 ^= 8.56, *P *= 0.003, OR = 1.5). No difference in the transmission of any alleles of the -839C/T polymorphism to ADHD was found in either of the two populations (Table 2).

**Table 1:** TDT Analysis of the DAT1 Promoter -67A/T Polymorphism

	UK samples Allele		Taiwanese samples Allele		Combined samples Allele	
	A	T	A	T	A	T

Transmitted	37	43	45	81	82	124
Non-transmitted	43	37	81	45	124	82
χ^2^, df (*P*-value)	0.45, 1 df (0.502)		10.43, 1 df (0.001)		8.56, 1 df (0.003)	

**Table 2:** TDT Analysis of the DAT1 Promoter -839C/T Polymorphism

	UK samples Allele		Taiwanese samples Allele		Combined samples Allele	
	C	T	C	T	C	T
Transmitted	50	49	40	55	90	104
Non-transmitted	49	50	55	40	104	90
χ^2^, df (*P*-value)	0.01, 1 df (0.919)		2.38, 1 df (0.123)		1.01, 1 df (0.315)	

Haplotype analysis revealed that the haplotype -67T/-839T was significantly over-transmitted to the Taiwanese ADHD probands and combined UK and Taiwanese samples (χ^2 ^= 12.47, *P *= 0.0004 in Taiwanese samples; χ^2 ^= 8.49, *P *= 0.004 in combined samples), while haplotype -67A/-839T was significantly under-transmitted to Taiwanese ADHD probands and combined samples (χ^2 ^= 13.13, *P *= 0.0003 in Taiwanese samples; χ^2 ^= 10.58, *P *= 0.001 in combined samples) (Table 3).

**Table 3:** Results of the TDT Analysis of the Haplotypes for -67A/T and -839C/T Polymorphisms

	**Haplotype**	**Transmitted**	**Freq-T**	**Non-transmitted**	**Freq-NT**	**χ^2^**	***P*-value**
TW	-67T/-839T	60	0.69	22	0.25	12.47	0.0004
	-67T/-839C	9	0.1	17	0.2	2.5	0.11
	-67A/-839T	4	0.05	25	0.29	13.1	0.0003
	-67A/-839C	14	0.16	23	0.26	1.61	0.02

UK	-67T/-839T	20	0.42	20	0.43	0	1.00
	-67T/-839C	2	0.04	5	0.12	1.29	0.26
	-67A/-839T	2	0.04	1	0.02	0.34	0.56
	-67A/-839C	24	0.5	20	0.43	0.28	0.60

Combined samples	-67T/-839T	80	0.59	42	0.32	8.49	0.004
	-67T/-839C	11	0.08	22	0.17	3.67	0.06
	-67A/-839T	6	0.05	26	0.19	10.58	0.001
	-67A/-839C	38	0.28	43	0.32	0.23	0.63

## Discussion

In this study, we set out to investigate previously reported findings of association between the two promoter polymorphisms of the DAT1gene in two independent samples of ADHD probands from UK and Taiwan. We found a significant over-transmission of the -67T allele to ADHD combined subtype cases in Taiwanese population and the two combined samples from UK and Taiwan. Our study replicates a finding conducted by Ohadi et al. [29], who found that the T allele of -67A/T polymorphism was over-represented (around 1.6 fold excess) in the ADHD probands compared to controls (χ^2 ^= 14.50, *P *< 0.001) in an Iranian sample. We did not observe any association with the -839C/T polymorphism and ADHD in either of our samples, and were thus unable to replicate the findings by Brookes et al [28] and Genro et al. [20]. In this study there were no effects at all on the two markers in the UK samples. However, haplotype analysis demonstrated significant preferential transmission of the -67T/-839T haplotype in the Taiwanese ADHD sample and the combined UK and Taiwanese samples. In addition, haplotypes -67A/-839T were under-transmitted to Taiwanese ADHD probands and combined samples.

## Conclusion

In conclusion, in this study we used family-based ADHD data in the UK and Taiwanese population to test for an association between two SNP variants in the promoter region of DAT1 and susceptibility to the disorder. Our findings support the notion that genetic variation in the promoter region of DAT1 might be a risk factor in the development of ADHD, particularly in the Taiwanese sample studied. Whilst further studies are needed to confirm these findings, our data suggest that association studies of DAT1 in ADHD should not be limited to variation at the 3' end of the gene, which has been the traditional focus for aetiological studies.

## Competing interests

The authors declare that they have no competing interests.

## Authors' contributions

XX selected the SNPs, performed genotyping, genetic analysis and drafted the manuscript. JM supervised sample preparation and revised the manuscript. BS assisted in the genotyping for the study. CKC, YSH and YYW provided the Taiwanese DNA samples and clinical data. PA supervised the study and revised the paper. All authors contributed to the final critical revision of the manuscript.

## Pre-publication history

The pre-publication history for this paper can be accessed here:


